# Thyroid dysfunction and its association with dyslipidaemia and dysglycaemia in adults: a cross-sectional study of primary-care laboratory records (2019–2025)

**DOI:** 10.1136/bmjopen-2026-125212

**Published:** 2026-09-18

**Authors:** Hülya Doğan Tiryaki, Kevser Erdoğan, Osman Kurt, Nefise Şeker, Osman Küçükkelepçe

**Affiliations:** 1 Department of Public Health, Gaziantep University, Gaziantep, Türkiye; 2 Department of Public Health, Antalya Training and Research Hospital, Antalya, Türkiye; 3 Department of Public Health, İnönü University Faculty of Medicine, Malatya, Türkiye; 4 Department of Public Health, Şanlıurfa Provincial Health Directorate, Şanlıurfa, Türkiye; 5 Department of Public Health, Gülhane Faculty of Medicine, University of Health Sciences, Ankara, Türkiye

**Keywords:** Epidemiology, Thyroid disease, Lipid disorders, DIABETES & ENDOCRINOLOGY, Primary Care, Cross-Sectional Studies

## Abstract

**Abstract:**

**Objectives:**

To determine the prevalence of thyroid dysfunction and its association with dyslipidaemia and dysglycaemia in adults.

**Design:**

Cross-sectional study.

**Setting:**

Primary-care laboratory records, Antalya, Türkiye, 2019–2025.

**Participants:**

Adults aged ≥18 years with a serum thyrotropin (TSH) measurement; the first eligible record per person was analysed (n=1 424 031).

**Primary and secondary outcome measures:**

Thyroid function status, classified from TSH and free thyroxine as euthyroid, subclinical or overt hypothyroidism and subclinical or overt hyperthyroidism; dyslipidaemia and dysglycaemia and their association with thyroid status, assessed by χ^2^ test, OR and multivariable logistic regression adjusted for age and sex, with E-value, calendar-year and interaction sensitivity analyses.

**Results:**

Among 1 316 090 adults with a complete thyroid assessment, 11.1% had thyroid dysfunction: subclinical hypothyroidism 7.97%, overt hypothyroidism 0.78%, subclinical hyperthyroidism 1.85% and overt hyperthyroidism 0.49%. Dysfunction was more frequent in women and increased with age. Correlations between TSH and lipid or glucose parameters were statistically significant but negligible in magnitude (Spearman rho 0.021 to 0.046 for lipids; −0.055 for glycated haemoglobin). Dyslipidaemia prevalence rose across hypothyroid categories, from 72.8% in euthyroid to 84.8% in overt hypothyroidism. Hypothyroidism was associated with dyslipidaemia (univariable OR 1.31 (1.29–1.33); age- and sex-adjusted OR 1.29 (1.27–1.31); p<0.001) and, more weakly, with dysglycaemia (adjusted OR 1.06 (1.04–1.07)). The adjusted association was unchanged after additional adjustment for calendar year, was consistent across years, and had an E-value of 1.52.

**Conclusions:**

Thyroid dysfunction—predominantly subclinical hypothyroidism—was common in this tested primary-care population and was associated with dyslipidaemia independent of age and sex. The association was modest, and residual confounding by unmeasured factors such as body mass index, thyroid medication and comorbidity cannot be excluded; the findings support attention to lipid status in adults with hypothyroidism but do not establish causality.

**STRENGTHS AND LIMITATIONS OF THIS STUDY**

- This very large primary-care laboratory sample of more than 1.4 million adults, tested in a single centralised laboratory, provides precise estimates of thyroid dysfunction and its metabolic correlates in a routinely tested population.
- Thyroid status was classified using both thyrotropin and free thyroxine (FT4), with a data-derived FT4 reference interval, and physiological plausibility limits were applied.
- The cross-sectional design cannot establish causality or temporal sequence, and thyroid status was based on a single measurement without antibody testing or confirmatory repeat sampling.
- Participants were adults tested in routine primary care rather than a random population sample, so selection bias is possible and findings generalise to the tested population rather than the general community.
- Models adjusted only for age and sex; information on body mass index, thyroid medication, diet, iodine status and comorbidity was unavailable, so residual confounding is likely, although the E-value (1.52) and calendar-year analyses indicate the association is not readily explained by weak unmeasured confounding or period effects.

## Introduction

 Thyroid dysfunction is among the most common endocrine disorders worldwide. It is defined biochemically by an abnormal serum thyrotropin (TSH), and its severity is graded using free thyroxine (FT4): subclinical hypothyroidism denotes a raised TSH with a normal FT4, overt hypothyroidism a raised TSH with a low FT4, and the corresponding suppressed-TSH states define subclinical and overt hyperthyroidism.^1^ Reported prevalence varies substantially with age, sex, iodine status, ethnicity and the diagnostic thresholds applied, but subclinical hypothyroidism is consistently the most frequent abnormality detected on screening and is more common in women and in older people.^2–5^

Thyroid hormones are central regulators of lipid metabolism. They upregulate hepatic low-density lipoprotein (LDL) receptor expression and modulate cholesterol synthesis and biliary clearance; in the hypothyroid state, reduced LDL-receptor activity impairs cholesterol clearance and raises total and LDL cholesterol, while thyroid hormone and TSH additionally influence triglyceride and high-density lipoprotein (HDL) handling.^6^ Consequently, even mild thyroid hypofunction can shift the lipid profile in an atherogenic direction, providing a mechanistic basis for an association between thyroid dysfunction and dyslipidaemia.

These metabolic effects are clinically important because subclinical and overt thyroid dysfunction have been associated with adverse cardiovascular outcomes. Large individual-participant-data analyses have linked subclinical hypothyroidism to coronary heart disease events and mortality, and subclinical thyroid dysfunction to heart failure, although the strength of these associations varies with age and the degree of TSH elevation and is not uniform across cohorts.^7–10^

Thyroid function is also intertwined with glucose homeostasis. Thyroid hormones influence insulin secretion and sensitivity and hepatic glucose output, and thyroid dysfunction and disorders of glucose metabolism frequently coexist; subclinical thyroid dysfunction has been examined as a potential correlate of incident diabetes, although the evidence is mixed.^11 12^

Despite the clinical importance of these relationships, large, contemporary, real-world estimates of the prevalence of thyroid dysfunction and its association with dyslipidaemia and dysglycaemia remain limited; many population studies are comparatively small, are confined to selected groups, or predate current practice.^13 14^ Primary-care laboratory records, which capture routine thyroid, lipid and glucose testing in large clinical populations, offer an opportunity to characterise these relationships at scale and to inform whether metabolic assessment should accompany the detection of thyroid dysfunction in routine care.^15^

Using 7 years of primary-care laboratory records from a large Turkish province, we aimed to (1) determine the prevalence of thyroid dysfunction and its categories in adults; (2) describe its distribution by sex and age and (3) quantify its association with dyslipidaemia and dysglycaemia, including the association of hypothyroidism with dyslipidaemia after adjustment for age and sex, together with sensitivity analyses addressing unmeasured confounding, calendar-time trends and effect modification.

## Methods

### Study design, setting and population

This cross-sectional study was based on the electronic laboratory records of adults who attended family health centres (primary care) in Antalya, a large province on the Mediterranean coast of Türkiye, and whose blood samples were analysed centrally at the province’s public-health laboratory—a tier L1 laboratory within Türkiye’s national public-health laboratory network—between 1 January 2019 and 31 December 2025, so that uniform analytical methods were applied throughout and interlaboratory variability was minimised. Adults aged 18 years or older with at least one serum TSH measurement were eligible. Because the aim was a cross-sectional description and to avoid over-representing individuals with repeated testing, the first eligible record per person during the study period was taken as the unit of analysis; individuals were linked across time through a de-identified person key. No sample-size calculation was performed, as all eligible records were included. The study is reported in accordance with the Strengthening the Reporting of Observational Studies in Epidemiology (STROBE) and RECORD recommendations.^16 17^

### Thyroid function classification

**Figure 1:**
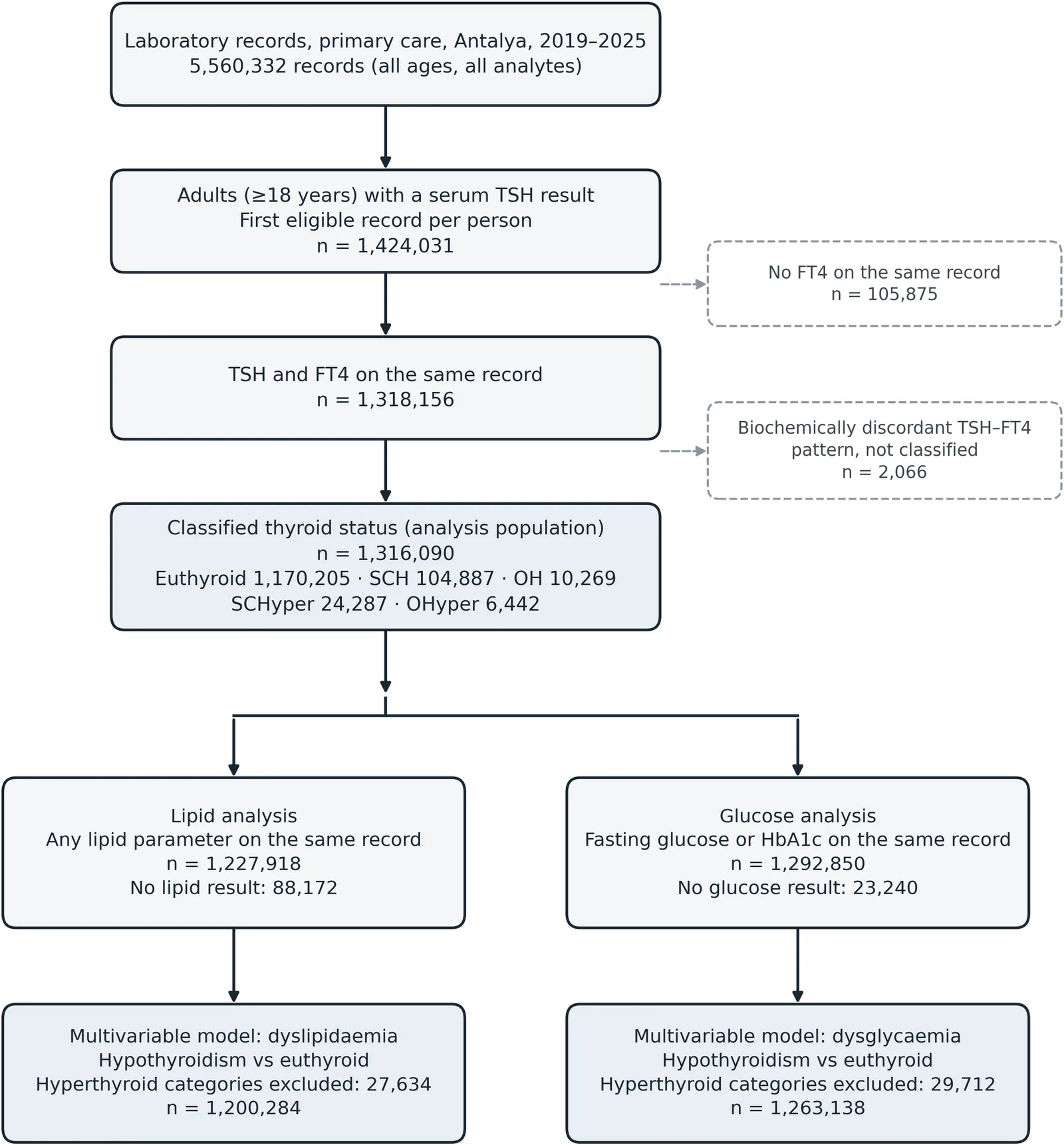
Flow of participants through the study. Numbers are shown at each step from the source laboratory records to the analysis population and to the outcome-specific analyses. FT4, free thyroxine; HbA1c, glycated haemoglobin; OH, overt hypothyroidism; OHyper, overt hyperthyroidism; SCH, subclinical hypothyroidism; SCHyper, subclinical hyperthyroidism; TSH, thyrotropin.

Thyroid status was classified from paired TSH and FT4 results obtained on the same record. A TSH concentration of 0.4–4.0 mIU/L was taken as the reference interval, consistent with widely used clinical thresholds.^15^ Because assay-specific FT4 reference intervals may not be transferable, the FT4 reference interval was derived directly from the study population using an indirect (data-mining) approach in line with CLSI EP28 guidance: among euthyroid individuals (TSH 0.4–4.0 mIU/L), the 2.5th and 97.5th centiles of FT4 defined the reference limits,^18^ yielding 0.63–1.16 ng/dL. Participants with both TSH and FT4 were classified as euthyroid (TSH 0.4–4.0 mIU/L); subclinical hypothyroidism (TSH>4.0 mIU/L with FT4 within the reference interval); overt hypothyroidism (TSH>4.0 mIU/L with FT4 below the reference interval); subclinical hyperthyroidism (TSH<0.4 mIU/L with FT4 within the reference interval) or overt hyperthyroidism (TSH<0.4 mIU/L with FT4 above the reference interval). Rare biochemically discordant combinations (eg, a raised TSH with a raised FT4) were not assigned to these categories and were excluded from the analysis population (n=2066; figure 1).

### Metabolic outcomes

Lipid and glucose status were defined from the same record. Dyslipidaemia was defined, following the National Cholesterol Education Program Adult Treatment Panel III, as the presence of any of total cholesterol ≥200 mg/dL, LDL cholesterol ≥130 mg/dL, HDL cholesterol <40 mg/dL in men or <50 mg/dL in women or triglycerides ≥150 mg/dL.^19^ Dysglycaemia was defined as a fasting glucose ≥100 mg/dL or a glycated haemoglobin (HbA1c) ≥5.7%, encompassing the pre-diabetes and diabetes ranges in accordance with American Diabetes Association criteria.^20^ Because routine testing is indication-driven, not every record contained a full lipid panel or both glucose measures; dyslipidaemia was assigned from the lipid parameters available on the record and dysglycaemia from whichever glucose measure was available, and individuals with no lipid (or no glucose) result on the index record were excluded from the corresponding analysis only.

### Data source, integration and quality control

Monthly laboratory extracts were merged into a single dataset. Direct personal identifiers (name, national identity number and physician details) were removed at source, and repeat attendances were linked through a de-identified person key derived from the identity number. Physiological plausibility limits were applied to every analyte to exclude recording and unit errors (eg, TSH 0.005–100 mIU/L, FT4 0.1–8 ng/dL, total cholesterol 50–500 mg/dL, LDL 20–400 mg/dL, HDL 10–150 mg/dL, triglycerides 20–2000 mg/dL, glucose 40–600 mg/dL and HbA1c 3%–20%); values outside these ranges were set to missing. The first eligible record per person was then selected for the cross-sectional analysis.

### Missing data

Missingness arose only from tests not requested on the index record, not from unrecorded results. Of 1 424 031 adults with a TSH result, 105 875 (7.4%) had no FT4 on the same record and could not be classified; of the 1 316 090 classified adults, 88 172 (6.7%) had no lipid result and 23 240 (1.8%) had no glucose result. Complete-case analysis was used for each outcome; no imputation was performed, because the analytes missing were those not clinically requested rather than values lost at random. The flow of participants through each analysis, with the numbers excluded at each step, is shown in figure 1.

### Statistical analysis

Analyses were performed with IBM SPSS Statistics V.22 and independently re-run in Python 3.12 (pandas 3.0, SciPy and statsmodels), from which all reported estimates derive. Continuous variables, which were non-normally distributed, were summarised as median and IQR and categorical variables as counts and percentages. Lipid and glucose parameters were compared across the five thyroid categories using the Kruskal–Wallis test, and categorical variables using the Pearson χ^2^ test. The strength of association between TSH and each lipid and glucose parameter was assessed with the Spearman rank correlation coefficient, interpreted primarily by magnitude given the very large sample. The associations of thyroid status with dyslipidaemia and dysglycaemia were expressed as OR with 95% CIs. A multivariable binary logistic regression model estimated the association of hypothyroidism (subclinical or overt, vs euthyroid) with dyslipidaemia, adjusting for age (per 10 years) and sex; age and sex were selected a priori as established determinants of both thyroid dysfunction and dyslipidaemia and as the only potential confounders available in the laboratory record. A parallel model was fitted for dysglycaemia, and category-specific models (each thyroid category vs euthyroid) were fitted for both outcomes to examine whether crude differences persisted after adjustment.

Four sensitivity analyses were performed. First, to quantify robustness to unmeasured confounding, we calculated the E-value for the adjusted association of hypothyroidism with dyslipidaemia; because the outcome is common (prevalence 73.2% in the model population), the OR was first converted to an approximate risk ratio by taking its square root, as recommended.^21^ Second, to assess temporal trends, prevalence estimates were tabulated by calendar year of the index record, the adjusted model was refitted with calendar year as an additional covariate, and year-specific adjusted ORs were estimated. Third, effect modification by sex and by age was examined by adding multiplicative interaction terms and by stratified models. Fourth, dyslipidaemia and dysglycaemia were compared with the euthyroid group for each thyroid category with adjustment for age and sex. A two-sided p value<0.05 was considered statistically significant, but given the sample size, interpretation emphasised effect sizes and CIs over p values.

### Patient and public involvement

None. Patients or the public were not involved in the design, conduct, reporting or dissemination plans of this research, which used de-identified retrospective laboratory records.

## Results

### Population and prevalence of thyroid dysfunction

From 5 560 332 laboratory records, 1 424 031 adults with a TSH measurement were identified (mean age 44.2 (SD 16.7) years; 60.5% women). Of these, 1 318 156 also had an FT4 result on the same record, and after exclusion of 2066 biochemically discordant profiles, 1 316 090 adults with a classified thyroid status formed the analysis population (figure 1). In this population 11.1% had thyroid dysfunction. Subclinical hypothyroidism was the most frequent abnormality (7.97%), followed by subclinical hyperthyroidism (1.85%), overt hypothyroidism (0.78%) and overt hyperthyroidism (0.49%); 88.92% were euthyroid (table 1).

**Table 1:** Characteristics of the study population

Characteristic	Value
Adults with a TSH measurement, n	1 424 031
Age, years, mean (SD)	44.2 (16.7)
Women, n (%)	861 013 (60.5)
Thyroid status, n (%)*	n=1 316 090
Euthyroid	1 170 205 (88.92)
Subclinical hypothyroidism	104 887 (7.97)
Overt hypothyroidism	10 269 (0.78)
Subclinical hyperthyroidism	24 287 (1.85)
Overt hyperthyroidism	6442 (0.49)
Laboratory, median (IQR)†	
TSH, mIU/L (n=1 424 031)	1.8 (1.2–2.7)
Free T4, ng/dL (n=1 318 156)	0.9 (0.8–0.9)
Total cholesterol, mg/dL (n=1 303 492)	200.0 (171.0–233.0)
LDL cholesterol, mg/dL (n=1 269 563)	123.0 (100.0–149.0)
HDL cholesterol, mg/dL (n=1 255 461)	50.4 (43.2–59.0)
Triglycerides, mg/dL (n=1 306 845)	112.0 (78.0–164.0)
Fasting glucose, mg/dL (n=1 391 782)	92.0 (85.0–101.0)
HbA1c, % (n=1 004 484)	5.7 (5.4–6.1)

* Thyroid status is shown for the 1 316 090 adults with both TSH and FT4 on the index record and a classifiable TSH–FT4 pattern (figure 1); percentages use this denominator.

† Laboratory values are summarised among adults with the respective test on the index record (n shown in each row).

HbA1c, glycated haemoglobin; HDL, high-density lipoprotein; LDL, low-density lipoprotein; TSH, thyrotropin.

### Distribution by sex and age

**Figure 2:**
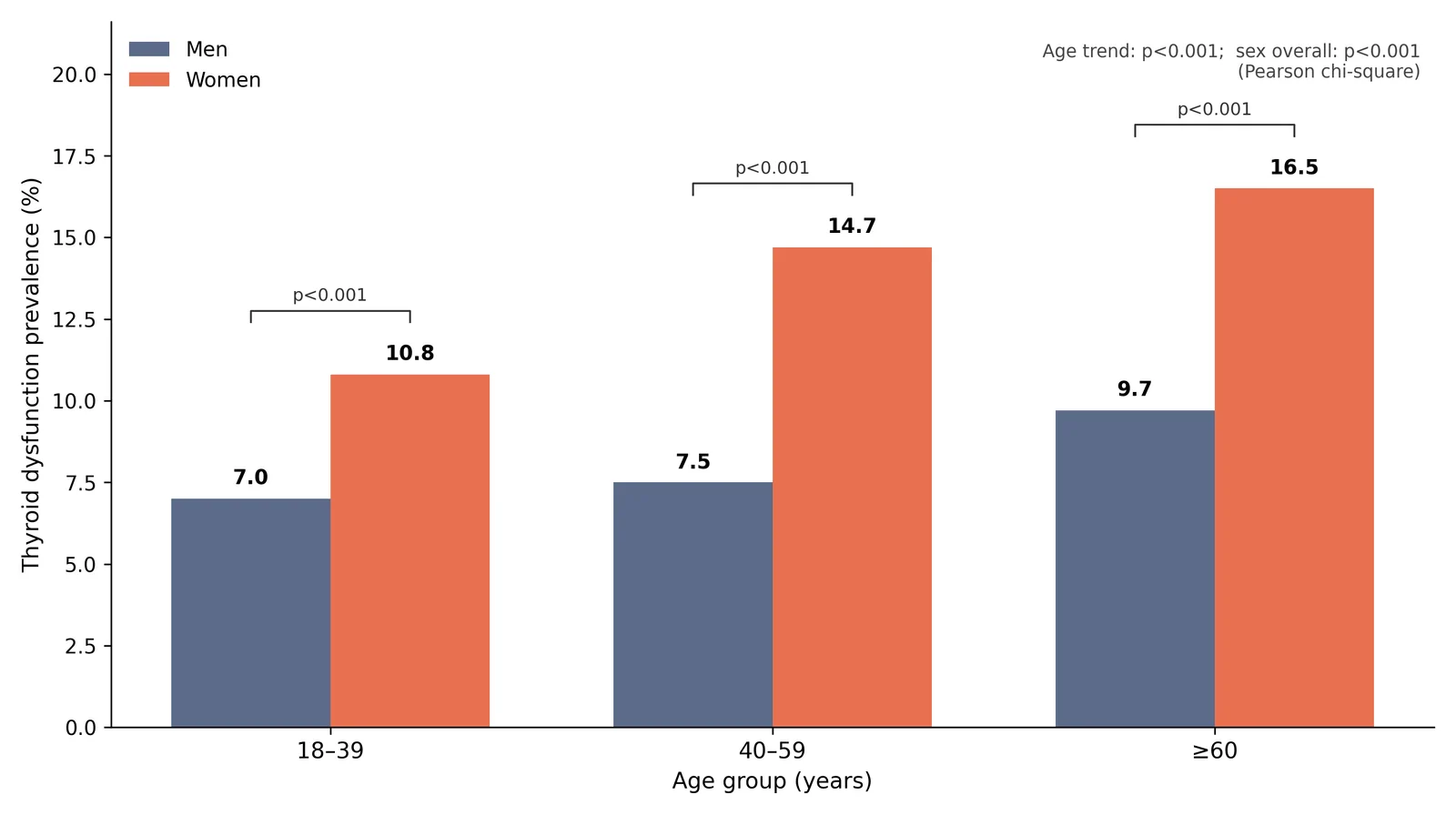
Prevalence of thyroid dysfunction by age group and sex. Brackets show Pearson χ^2^ p values for the sex difference within each age group; overall p values for the age trend and for sex are given in the panel.

Thyroid dysfunction was more frequent in women than men (13.2% vs 7.8%; p<0.001) and increased with age, from 9.5% at 18–39 years to 13.4% at ≥60 years (p<0.001); the sex difference was present in every age group (table 2, figure 2).

**Table 2:** Prevalence of thyroid dysfunction by sex and age group

Variable	Category	N	Thyroid dysfunction, %	P value
Sex	Women	796 725	13.2	<0.001
	Men	519 365	7.8	
Age group	18–39 years	563 476	9.5	<0.001
	40–59 years	486 982	11.6	
	≥60 years	265 632	13.4	

Among 1 316 090 adults with a classified thyroid status. Comparisons used the Pearson χ2 test. Sex-specific prevalence within each age group is shown in figure 2 (all p<0.001).

### Thyroid status and metabolic parameters

**Figure 3:**
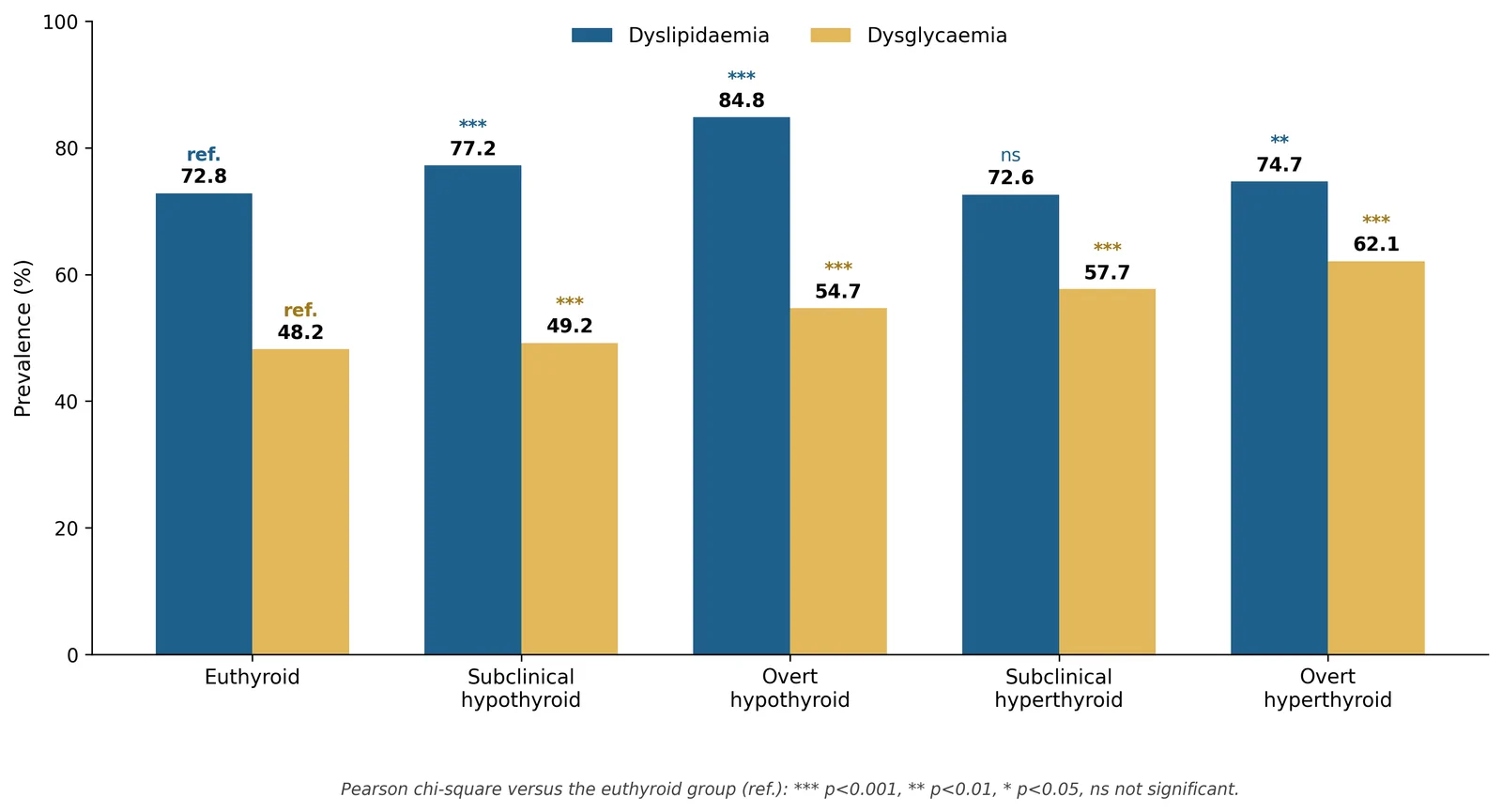
Prevalence of dyslipidaemia and dysglycaemia across thyroid function categories. Asterisks indicate Pearson χ^2^ comparisons with the euthyroid group (***p<0.001, **p<0.01, *p<0.05, ns, not significant).

Lipid and glucose parameters differed across thyroid categories (all p<0.001, table 3), with total and LDL cholesterol and triglycerides highest in overt hypothyroidism and fasting glucose and HbA1c highest in the hyperthyroid categories. TSH showed statistically significant but negligible correlations with total cholesterol (Spearman rho 0.036), LDL cholesterol (0.021), HDL cholesterol (0.026) and triglycerides (0.046), and weak negative correlations with fasting glucose (−0.052) and HbA1c (−0.055) (all p<0.001); these coefficients indicate that TSH explains well under 1% of the variance in any parameter. Dyslipidaemia prevalence rose across hypothyroid categories, from 72.8% in euthyroid adults to 77.2% in subclinical and 84.8% in overt hypothyroidism, whereas it did not differ from euthyroid adults in subclinical hyperthyroidism (72.6%; p=0.59) (figure 3). Dysglycaemia was more frequent in every dysfunction category than in euthyroid adults (48.2%), most markedly in subclinical (57.7%) and overt (62.1%) hyperthyroidism (figure 3).

**Table 3:** Lipid and glucose parameters (median (IQR), mg/dL unless stated) across thyroid function categories

Parameter	Euthyroid	Subclinical hypothyroidism	Overt hypothyroidism	Subclinical hyperthyroidism	Overt hyperthyroidism	P value
Total cholesterol	199.0 (170.0–232.0)	206.0 (175.0–240.0)	221.0 (189.0–255.0)	199.0 (171.0–231.0)	194.0 (164.0–225.0)	<0.001
LDL cholesterol	123.0 (99.0–149.0)	126.0 (102.0–154.0)	137.0 (112.0–165.0)	123.0 (99.0–148.0)	118.0 (95.0–144.0)	<0.001
HDL cholesterol	50.3 (43.1–59.0)	51.4 (44.0–60.0)	52.4 (45.0–61.4)	51.0 (44.0–59.8)	50.0 (43.0–59.0)	<0.001
Triglycerides	111.0 (77.0–163.0)	119.0 (84.0–174.0)	136.0 (94.0–197.0)	109.0 (78.0–155.0)	106.0 (78.0–145.0)	<0.001
Fasting glucose	92.0 (85.0–101.0)	92.0 (85.0–101.0)	93.0 (85.0–102.0)	95.0 (87.0–106.0)	96.0 (88.0–109.0)	<0.001
HbA1c, %	5.7 (5.4–6.1)	5.7 (5.4–6.1)	5.8 (5.5–6.2)	5.8 (5.5–6.2)	5.8 (5.5–6.3)	<0.001

Comparisons used the Kruskal–Wallis test. Spearman correlations of TSH with each parameter (all p<0.001): total cholesterol 0.036, LDL 0.021, HDL 0.026, triglycerides 0.046, fasting glucose −0.052, HbA1c −0.055.

HbA1c, glycated haemoglobin; HDL, high-density lipoprotein; LDL, low-density lipoprotein.

### Association of hypothyroidism with dyslipidaemia and dysglycaemia

**Figure 4:**
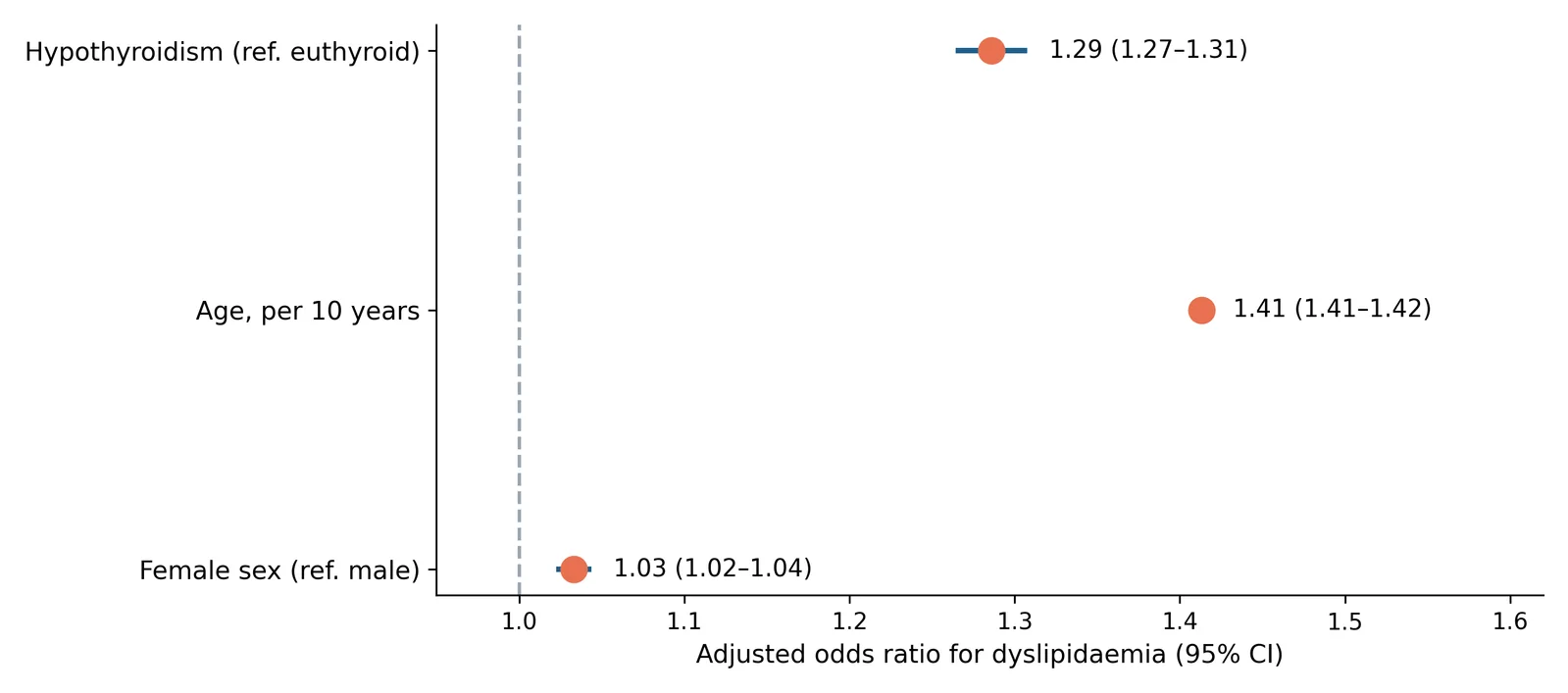
Adjusted ORs with 95% CIs for dyslipidaemia (multivariable logistic regression, n=1 200 284). The dashed line indicates the null value (OR=1).

Among 1 200 284 adults with hypothyroidism or euthyroidism and a lipid result (the 27 634 adults with hyperthyroidism were not included in this comparison; figure 1), dyslipidaemia was more frequent in hypothyroid than euthyroid adults (univariable OR 1.31 (1.29–1.33); p<0.001). After adjustment for age and sex, the association persisted (adjusted OR 1.29 (1.27–1.31); p<0.001) alongside strong associations with age (adjusted OR 1.41 (1.41–1.42) per 10 years) and a minimal association with female sex (adjusted OR 1.03 (1.02–1.04)) (table 4, figure 4). Hypothyroidism was also associated with dysglycaemia (n=1 263 138; univariable OR 1.06 (1.05–1.08); adjusted OR 1.06 (1.04–1.07)), an association an order of magnitude weaker than that of age (adjusted OR 2.01 (2.01–2.02) per 10 years).

**Table 4:** Association of thyroid status with dyslipidaemia and dysglycaemia: main, adjusted and sensitivity analyses

Analysis	N (dyslipidaemia/dysglycaemia)	Dyslipidaemia, OR (95% CI)	Dysglycaemia, OR (95% CI)
Panel A: hypothyroidism (subclinical or overt) vs euthyroid			
Univariable	1 200 284/1 263 138	1.31 (1.29 to 1.33)	1.06 (1.05 to 1.08)
Adjusted for age and sex	1 200 284/1 263 138	1.29 (1.27 to 1.31)	1.06 (1.04 to 1.07)
Age, per 10 years (same models)		1.41 (1.41 to 1.42)	2.01 (2.01 to 2.02)
Female sex (same models)		1.03 (1.02 to 1.04)	0.69 (0.69 to 0.70)
Adjusted for age, sex and calendar year	1 200 284	1.29 (1.27 to 1.31)	—
E-value for the adjusted association (point estimate; lower confidence limit)		1.52; 1.50	—
Panel B: hypothyroidism vs euthyroid, dyslipidaemia, stratified			
Women (adjusted for age)	704 002	1.29 (1.27 to 1.31)	—
Men (adjusted for age)	496 282	1.20 (1.17 to 1.24)	—
Interaction hypothyroidism×female sex		1.06 (1.03 to 1.10)	—
18–39 years (adjusted for age and sex)	487 514	1.24 (1.21 to 1.26)	—
40–59 years (adjusted for age and sex)	462 744	1.35 (1.31 to 1.39)	—
≥60 years (adjusted for age and sex)	250 026	1.31 (1.26 to 1.36)	—
Interaction hypothyroidism×age (per 10 years)		1.07 (1.06 to 1.08)	—
Panel C: each thyroid category vs euthyroid, adjusted for age and sex			
Subclinical hypothyroidism	1 227 918/1 292 850	1.25 (1.23 to 1.27)	1.05 (1.04 to 1.07)
Overt hypothyroidism		1.82 (1.72 to 1.93)	1.12 (1.07 to 1.17)
Subclinical hyperthyroidism		0.73 (0.70 to 0.75)	0.92 (0.89 to 0.95)
Overt hyperthyroidism		0.73 (0.69 to 0.78)	0.98 (0.92 to 1.04)

All p<0.001 (Wald test) except overt hyperthyroidism vs euthyroid for dysglycaemia (p=0.48); the interaction terms had p<0.001 (sex) and p<0.001 (age). Hypothyroidism denotes subclinical or overt hypothyroidism; adults with hyperthyroidism were excluded from Panels A and B. Panel C models include all five categories with euthyroid as reference. For comparison, the univariable ORs for dysglycaemia in subclinical and overt hyperthyroidism were 1.47 (1.43–1.51) and 1.76 (1.68–1.86), respectively (see text). The E-value was calculated after converting the OR to an approximate risk ratio (square root) because the outcome is common.

### Sensitivity analyses

The E-value for the adjusted association between hypothyroidism and dyslipidaemia was 1.52 (lower confidence limit 1.50): an unmeasured confounder would need to be associated with both hypothyroidism and dyslipidaemia by a risk ratio of at least 1.52, beyond the measured covariates, to explain away the association, and by at least 1.50 to shift its CI to include the null (table 4). Additional adjustment for calendar year left the estimate unchanged (adjusted OR 1.29 (1.27–1.31)), and year-specific adjusted ORs ranged from 1.24 to 1.32 with overlapping confidence intervals (table 5). The prevalence of thyroid dysfunction declined modestly over the period (11.6% in 2019 to 10.7% in 2025; p<0.001), accompanied by a marked change in the composition of the tested population, in which the proportion of women fell from 70.9% to 47.2% (table 5).

**Table 5:** Prevalence of thyroid dysfunction and metabolic abnormalities, and the adjusted association of hypothyroidism with dyslipidaemia, by calendar year of the index record

Year	N	Mean age, years	Women, %	Thyroid dysfunction, %	Subclinical hypothyroidism, %	Dyslipidaemia, %	Dysglycaemia, %	Hypothyroidism→dyslipidaemia, adjusted OR (95% CI)
2019	291 922	45.4	70.9	11.6	7.7	75.5	49.3	1.32 (1.27 to 1.36)
2020	146 861	43.7	68.6	11.8	8.4	76.4	44.6	1.30 (1.24 to 1.37)
2021	160 320	44.1	66.1	11.2	8.2	72.9	50.4	1.29 (1.24 to 1.35)
2022	181 873	45.0	59.5	11.1	8.1	73.1	49.0	1.30 (1.25 to 1.36)
2023	193 977	44.3	55.1	10.7	7.8	71.7	47.6	1.29 (1.24 to 1.34)
2024	168 758	42.8	51.2	10.3	7.7	71.5	47.1	1.24 (1.19 to 1.30)
2025	172 379	42.5	47.2	10.7	8.2	71.1	51.0	1.25 (1.20 to 1.30)

n refers to adults with a classified thyroid status whose index record fell in that year (records dated in the last days of December 2018 within the January 2019 extract, n=13, are counted in 2019). Dyslipidaemia and dysglycaemia percentages are among adults with the respective test. Adjusted ORs are from year-specific models adjusted for age and sex (hypothyroid or euthyroid adults with a lipid result). χ2 test for difference in thyroid dysfunction prevalence across years: p<0.001.

Interaction terms for hypothyroidism with sex (interaction OR 1.06 (1.03–1.10)) and with age (1.07 (1.06–1.08) per 10 years) were statistically significant but small: the adjusted OR was 1.29 (1.27–1.31) in women and 1.20 (1.17–1.24) in men, and 1.24 (1.21–1.26), 1.35 (1.31–1.39) and 1.31 (1.26–1.36) at 18–39, 40–59 and ≥60 years, respectively (table 4). In category-specific models, dyslipidaemia was associated with subclinical (adjusted OR 1.25 (1.23–1.27)) and overt hypothyroidism (1.82 (1.72–1.93)) and inversely with subclinical (0.73 (0.70–0.75)) and overt (0.73 (0.69–0.78)) hyperthyroidism. For dysglycaemia, the crude excess in subclinical (univariable OR 1.47 (1.43–1.51)) and overt hyperthyroidism (1.76 (1.68–1.86)) was fully accounted for by age and sex (adjusted OR 0.92 (0.89–0.95) and 0.98 (0.92–1.04)), consistent with the older mean age of these groups (52.0 and 55.0 years vs 43.8 years in euthyroid adults), whereas small adjusted associations remained for subclinical (1.05 (1.04–1.07)) and overt hypothyroidism (1.12 (1.07–1.17)) (table 4).

## Discussion

In this cross-sectional analysis of more than 1.3 million adults with a classified thyroid status from primary-care laboratory records, thyroid dysfunction was present in 11.1%, was dominated by subclinical hypothyroidism, was more frequent in women and older adults, and was associated with dyslipidaemia independent of age and sex. Dyslipidaemia prevalence increased across the hypothyroid spectrum, reaching 84.8% in overt hypothyroidism, and the adjusted association was stable across seven calendar years and robust to weak-to-moderate unmeasured confounding.

The prevalence and pattern of thyroid dysfunction are consistent with previous population studies, in which subclinical hypothyroidism is the most common abnormality and rises with age and female sex.^2–4^ Our very large clinical sample adds precise, contemporary estimates from a Mediterranean setting and confirms these demographic gradients at scale in a routinely tested population.

The graded increase in dyslipidaemia across hypothyroid categories, and its persistence after adjustment for age and sex, are biologically coherent. Thyroid hormone deficiency reduces LDL-receptor-mediated clearance and raises total and LDL cholesterol, so even mild thyroid hypofunction can shift the lipid profile unfavourably.^6^ This mechanistic link, together with the associations between thyroid dysfunction and cardiovascular outcomes reported in collaborative cohorts, supports attention to lipid status in adults with hypothyroidism.^7–9^

The magnitude of the associations deserves emphasis. With more than a million observations, almost any difference reaches conventional significance, so p values are uninformative and effect sizes must guide interpretation. The correlations between TSH and individual lipid or glucose parameters were negligible (all |rho|<0.06), the adjusted OR for female sex was trivially above unity, and even the hypothyroidism–dyslipidaemia association, although consistent, was modest (adjusted OR 1.29) and an order of magnitude smaller than the effect of a decade of age. The near-identity of the crude (1.31) and adjusted (1.29) ORs reflects the limited confounding by age and sex specifically, not the absence of confounding in general. The E-value of 1.52 indicates that an unmeasured factor associated with both hypothyroidism and dyslipidaemia by a risk ratio of about 1.5 could fully account for the association^21^; adiposity is a plausible candidate, since obesity is associated with higher TSH and with an atherogenic lipid profile, and its omission is likely to have biased the association away from the null. Conversely, statin or levothyroxine treatment among adults already known to have dyslipidaemia or hypothyroidism would tend to attenuate the observed association. The direction of net bias is therefore uncertain, and the estimate should be read as an association in a tested population rather than a causal effect.

Hypothyroidism was also weakly associated with dysglycaemia, in keeping with the recognised, if complex, relationship between thyroid function and glucose metabolism,^11 12^ but age was the dominant determinant of dysglycaemia. The apparently higher dysglycaemia prevalence in the hyperthyroid categories, which did not follow a dose–response pattern across thyroid states, was entirely explained by the older age of these groups: after adjustment, subclinical hyperthyroidism was, if anything, inversely associated with dysglycaemia. This pattern is consistent with confounding by indication, since low TSH in older adults frequently reflects levothyroxine over-replacement or non-thyroidal illness rather than endogenous hyperthyroidism, and illustrates why unadjusted comparisons across thyroid categories should be interpreted cautiously.

Effect modification by sex and age was statistically detectable but small in absolute terms (adjusted ORs of 1.29 in women and 1.20 in men, and 1.24 to 1.35 across age groups); we do not consider these differences clinically actionable. Likewise, the modest decline in thyroid dysfunction prevalence over 2019–2025 coincided with a shift in the tested population from about 70% to under 50% women and is more plausibly a change in who was tested than in underlying disease frequency; the stability of the year-specific adjusted ORs argues against a period artefact in the main association.

### Generalisability

Our findings apply to adults who attended primary care and had thyroid function tested, not to the general population. Testing is indication-driven: people are tested because of symptoms, screening in pregnancy or chronic disease, or monitoring of known thyroid disease, and those who never attend are systematically different. Such selection may inflate the prevalence of thyroid dysfunction relative to the community and, if the reasons for testing are also related to lipid status, could bias the association in either direction. The predominance of women, particularly in the earlier years, and the shifting sex composition over time both reflect testing patterns rather than population structure. The estimates should therefore be regarded as descriptive of the tested primary-care population of one Mediterranean province; the centralised laboratory, standardised assays and large numbers support internal validity, but external validity to other settings depends on the similarity of testing practices and iodine status.

These findings have practical implications. Given the high frequency of subclinical hypothyroidism and its association with an adverse lipid profile, clinicians could reasonably consider lipid assessment in adults found to have thyroid dysfunction, and consider thyroid status when evaluating dyslipidaemia.^13 15^ Whether treating subclinical hypothyroidism improves lipid or cardiovascular outcomes remains uncertain and is beyond the scope of a cross-sectional study.

### Limitations

Several limitations warrant emphasis. First, the cross-sectional design precludes causal inference and cannot establish temporal sequence. Second, participants were a tested clinical population rather than a random sample, so selection bias is possible, as discussed above. Third, and most importantly, models adjusted only for age and sex. Body mass index, thyroid medication, lipid-lowering therapy, iodine status, diet, smoking and comorbidity were unavailable in the laboratory record; these are potential confounders, and the E-value indicates that a moderately strong unmeasured confounder could explain the association. Fourth, thyroid status was based on a single measurement without antibody testing or confirmatory repeat sampling, so transient abnormalities and non-thyroidal illness will have been misclassified as dysfunction. Fifth, dyslipidaemia and dysglycaemia were assigned from whichever parameters were requested, and fasting status could not be verified for glucose; complete-case analysis assumed that the availability of a test was unrelated to its result conditional on thyroid status, which cannot be confirmed. Finally, pooling 7 years of data assumes a stable relationship over time; the year-stratified analyses supported this assumption for the main association, but the composition of the tested population changed markedly over the period.

## Conclusion

Thyroid dysfunction, predominantly subclinical hypothyroidism, was common among adults tested in primary care and was associated with dyslipidaemia independent of age and sex, with a graded increase across the hypothyroid spectrum. The association was modest, consistent across calendar years and subgroups, and robust to weak unmeasured confounding, but the cross-sectional design and the absence of data on adiposity, medication and comorbidity mean that a causal effect cannot be inferred. Within these limits, the findings support integrated assessment of thyroid and lipid status in routine practice while underlining the need for longitudinal studies with fuller covariate data.

## Supplementary material

10.1136/bmjopen-2026-125212 online supplemental file 1

## Data Availability

Data are available upon reasonable request.
