# Supplementary material for: Thyroid dysfunction and its association with dyslipidaemia and dysglycaemia in adults: a cross-sectional study of primary-care laboratory records (2019–2025)
Source: BMJ Open. 2026 Sep 18;16(9):e125212. doi: 10.1136/bmjopen-2026-125212 (PMC13599846; doi:10.1136/bmjopen-2026-125212)
Supplement: Supplementary file 1 [file bmjopen-2026-125212.reviewer_comments.pdf]

PEER REVIEW HISTORY

BMJ Open publishes all reviews undertaken for accepted manuscripts. Reviewers are asked to complete a checklist review form and are provided with free text boxes to elaborate on their assessment. These free text comments are reproduced below.

ARTICLE DETAILS

Title (Provisional)

Thyroid dysfunction and its association with dyslipidaemia and dysglycaemia in adults: a cross-sectional study of primary-care laboratory records (2019-2025)

Authors

Doğan Tiryaki, Hülya; Erdoğan, Kevser; kurt, osman; Şeker, Nefise; Küçükkelepçe, Osman

---

VERSION 1 - REVIEW

**Reviewer**	**1**
**Name**	**Dack, Kyle**
**Affiliation**	**University of Bristol, MRC Integrative Epidemiology Unit**
**Date**	**28-Jul-2026**
**COI**	**None**

Statistical review:

The study has several strengths including the large sample size, consistent lab methods, and the manuscript has good adherence to STROBE. However, there are limitations which I think could be discussed in greater detail. (1) There is a risk of selection bias as this is not a random sample of the population but individuals who approached primary care. (2) Only age and sex were adjusted for - meaning confounding bias is a risk. (3) There may also be temporal trends hidden by pooling all the data into a single analysis. Limitations (1) and (2) are beyond the control of the authors, but these could be discussed in more detail, along with an evaluation of the potential impact on the results.

Abstract:

1. Line 33-34: To make the statistical presentation more consistent I recommend reporting the OR and 95% CI to the same number of decimal places and to avoid truncating zeros, e.g. OR 1.30 95% CI 1.28-1.32.

Methods:

2. What was the purpose of adjusting for age and sex, are these known confounders which affect both hypothyroidism and dyslipidaemia? If so, I think this could be directly stated so the multivariable models are interpreted appropriately.

3. Was there evidence of temporal trends? The study dates cover seven years and it is possible that within the population there was variation by year, especially for the overall prevalence estimates.

Results:

4. Similar to comment 1, I recommend that zeros are not truncated, as it will make the statistical presentation look more consistent. e.g. "OR 1.32, 95% CI 1.3–1.34" replacing 1.3 with 1.30.

5. Line 176. What was the reason for the multivariable model having a substantially lower N than the initially reported N? It could be helpful at the beginning of the results to report full exclusions, or consider a flow chart to show how the N evolved over the analysis.

6. "hypothyroidism was independently associated with dyslipidaemia" I suggest qualifying this statement more strongly - independent of age and sex, as there appear a number of important confounders which could not be adjusted for.

7. The statistical significance of the correlations/associations it not unexpected given the very large size of the dataset. Because of this, it would be informative to comment more on the magnitude of the correlations/associations, and their clinical importance. e.g. the correlations reported around line 1.65 are so small as to have little clinical importance, and similarly female sex in the adjusted model has minimal association, whereas age and dyslipidaemia have more notable associations.

8. Given the large sample size, did the authors consider evaluating the interaction between sex, age, and dyslipidaemia? I am unsure how clinically meaningful this would be, but from a statistical perspective it appears feasible to test in this dataset.

Discussion:

9. " Our very large, unselected clinical sample", " modest effect sizes are expected in an unselected population": while this phrasing may be accurate in that the study is based on electronic health records - it may give readers the impression that the results are representative of the general population. However, from what I can determine from the methods, these findings are based on individuals who approached primary care, which would typically be due to ill-health, pregnancy, or another concern. As such it may not be representative of the general population who did not attend primary care (potentially less healthy), and it would be worth discussing in more detail the generalizability of the results.

10. " important covariates—including thyroid medication, iodine status, diet and comorbidity— were unavailable" I think it could be more directly stated that these are potential confounders, and therefore could impact the reported associations. As this is a

significant limitation, the potential impact could be elaborated on - do the results risk underestimating/overestimating due to the lack of adjustment for these factors?

**Reviewer**	**2**
**Name**	**Zhang, Jin-an**
**Affiliation**	**Jinshan Hospital of Fudan University**
**Date**	**20-Aug-2026**
**COI**	**None**

This cross-sectional study analysed 1,426,416 adults with a TSH measurement and 1,318,078 with a complete thyroid assessment (TSH + FT4) from primary-care laboratory records in Antalya, Türkiye, 2019–2025. The authors report an 11.3% prevalence of thyroid dysfunction (dominated by subclinical hypothyroidism at 7.92%) and an adjusted OR of 1.3 (95% CI 1.28–1.32) for the association between hypothyroidism and dyslipidaemia after adjustment for age and sex. The dataset is impressively large, and the topic is clinically relevant. However, several methodological and statistical issues — most notably severe residual confounding from adjusting only for age and sex while the dataset is heavily confounded by indication, an unexplained 9% sample attrition between the complete-assessment sample (1,318,078) and the regression sample (1,199,764), and selective reporting of the dyslipidaemia–thyroid dose–response — mean the manuscript as currently presented does not support its principal conclusion. Major revision is required.

1、The multivariable model adjusts only for age and sex; the crude (1.32) → adjusted (1.30) shift is negligible, so residual confounding may fully explain the association. Please quantify via E-value/sensitivity analyses and state this limitation explicitly.

2、The complete thyroid assessment sample was n=1,318,078, but the regression sample included only n=1,199,764, a difference of 118,314 cases (≈9%) without any explanation. Please add a participant flow diagram and describe missing-data handling.

3、In Figure 3, the abnormal blood glucose rate in the subclinical hyperthyroidism group (57.7%) was higher than that in the overt hypothyroidism group (55.4%), which did not conform to the simple dose-response pattern. It is more likely that this was caused by confounding factors such as the selection of detection indicators. Please provide additional discussion and necessary analysis.

4、STROBE checklist, flow diagram, sensitivity analyses, and a Generalisability paragraph are missing. Please add them.

5、Abstract omits the univariable OR, dysglycaemia OR, and Spearman coefficients. Please add them.

6、Figure 2 has no P value, and Figure 3 has no group significance markers.

7、Figure 1 is exactly the same as Table 1. It is recommended to redesign or delete Figure 1.

VERSION 1 - AUTHOR RESPONSE

Reviewer 1 (Dr Kyle Dack, University of Bristol) — statistical review

General

Comment: The study has several strengths including the large sample size, consistent lab methods, and the manuscript has good adherence to STROBE. However, there are limitations which I think could be discussed in greater detail. (1) There is a risk of selection bias as this is not a random sample of the population but individuals who approached primary care. (2) Only age and sex were adjusted for - meaning confounding bias is a risk. (3) There may also be temporal trends hidden by pooling all the data into a single analysis. Limitations (1) and (2) are beyond the control of the authors, but these could be discussed in more detail, along with an evaluation of the potential impact on the results.

Response: We thank the reviewer for this balanced assessment and have acted on all three points. (1) Selection: a new Generalisability section explains that the sample is a tested primary-care population, why testing is indication-driven, how this may inflate prevalence and bias the association in either direction, and how the shifting sex composition of the tested population over time reflects testing patterns; the phrases 'unselected' and 'population-representative' have been removed throughout. (2) Confounding: we now name the unmeasured confounders explicitly, discuss the likely direction of bias for each (adiposity would bias away from the null; statin or levothyroxine treatment towards it), and quantify robustness with an E-value (1.52; lower confidence limit 1.50). (3) Temporal trends: we tabulate prevalence and the adjusted association by calendar year (new Table 5), refit the model with year as a covariate (adjusted OR unchanged at 1.29 (1.27–1.31)), and report year-specific adjusted ORs (range 1.24–1.32). Thyroid dysfunction prevalence declined modestly (11.6% to 10.7%), which we interpret as a change in the tested population (women fell from 70.9% to 47.2% of those tested) rather than in disease frequency; the main association was stable.

Changes made: New 'Generalisability' and 'Limitations' sections (pages 14–15); expanded fourth Discussion paragraph on magnitude and direction of confounding (page 13); new 'Sensitivity analyses' subsections in Methods (page 8) and Results (page 11); new Table 5 (page 22); Table 4 expanded (page 21).

1 (Abstract)

Comment: Line 33-34: To make the statistical presentation more consistent I recommend reporting the OR and 95% CI to the same number of decimal places and to avoid truncating zeros, e.g. OR 1.30 95% CI 1.28-1.32.

Response: Agreed. All ORs and CIs are now reported to two decimal places throughout the abstract, text, tables and figure 4 (e.g., adjusted OR 1.29 (1.27–1.31)).

Changes made: Abstract; Results; Tables 4 and 5; figure 4.

2 (Methods)

Comment: What was the purpose of adjusting for age and sex, are these known confounders which affect both hypothyroidism and dyslipidaemia? If so, I think this could be directly stated so the multivariable models are interpreted appropriately.

Response: We now state that age and sex were selected a priori as established determinants of both thyroid dysfunction and dyslipidaemia and as the only potential confounders available in the laboratory record, so that the adjusted estimate should be interpreted as independent of age and sex specifically, not of confounding in general.

Changes made: Methods, 'Statistical analysis' (page 8); Discussion, fourth paragraph (page 13).

3 (Methods)

Comment: Was there evidence of temporal trends? The study dates cover seven years and it is possible that within the population there was variation by year, especially for the overall prevalence estimates.

Response: Yes, and we now report it. By calendar year of the index record, thyroid dysfunction prevalence was 11.6%, 11.8%, 11.2%, 11.1%, 10.7%, 10.3% and 10.7% for 2019–2025 (chi-square $p<0.001$), while subclinical hypothyroidism stayed within 7.7–8.4%. Over the same period the proportion of women among tested adults fell from 70.9% to 47.2% and mean age from 45.4 to 42.5 years, so the modest decline in prevalence is most plausibly compositional. Critically for the main finding, the age- and sex-adjusted OR for hypothyroidism and dyslipidaemia was stable across years (1.24–1.32, overlapping CIs), and adding calendar year to the model left the pooled estimate unchanged (1.29 (1.27–1.31)). We note in Limitations that pooling assumes stability and that this assumption was supported for the association but that the tested population changed over time.

Changes made: New Table 5 (page 22); Methods 'Statistical analysis' (page 8); Results 'Sensitivity analyses' (page 11); Discussion (pages 13–15).

4 (Results)

Comment: Similar to comment 1, I recommend that zeros are not truncated, as it will make the statistical presentation look more consistent. e.g. "OR 1.32, 95% CI 1.3–1.34" replacing 1.3 with 1.30.

Response: Done throughout (see response to comment 1).

Changes made: Throughout.

5 (Results)

Comment: Line 176. What was the reason for the multivariable model having a substantially lower N than the initially reported N? It could be helpful at the beginning of the results to report full exclusions, or consider a flow chart to show how the N evolved over the analysis.

Response: We have added a flow chart as the new figure 1 (replacing the previous figure 1, which duplicated Table 1, as also suggested by Reviewer 2). It shows: 5,560,332 source records → 1,424,031 adults with a TSH result (first record per person) → 1,318,156 with FT4 on the same record (105,875 excluded) → 1,316,090 with a classifiable thyroid status (2,066 discordant excluded) → 1,227,918 with a lipid result and 1,292,850 with a glucose result → 1,200,284 and 1,263,138 in the hypothyroid-versus-euthyroid models (hyperthyroid categories excluded). The opening Results paragraph now walks through these numbers, and the reason for the smaller model N (missing lipid results plus exclusion of hyperthyroid adults from a hypothyroid-versus-euthyroid comparison) is stated explicitly.

Changes made: New figure 1 and legend; Results first paragraph (page 9) and 'Association…' paragraph (page 10); new 'Missing data' subsection (page 7).

6 (Results)

Comment: "hypothyroidism was independently associated with dyslipidaemia" I suggest qualifying this statement more strongly - independent of age and sex, as there appear a number of important confounders which could not be adjusted for.

Response: Agreed. 'Independently associated' has been replaced throughout by 'associated … independent of age and sex' (or 'after adjustment for age and sex'), including in the abstract, results, discussion and conclusion.

Changes made: Abstract (page 2); Results (page 10); Discussion (pages 12–15).

7 (Results)

Comment: The statistical significance of the correlations/associations it not unexpected given the very large size of the dataset. Because of this, it would be informative to comment more on the magnitude of the correlations/associations, and their clinical importance. e.g. the correlations reported around line 1.65 are so small as to have little clinical importance, and similarly female sex in the adjusted model has minimal association, whereas age and dyslipidaemia have more notable associations.

Response: We fully agree and have added a dedicated Discussion paragraph on magnitude. It states that with more than a million observations p values are uninformative; that the TSH correlations are negligible (all |rho| <0.06, explaining well under 1% of variance); that the adjusted OR for female sex (1.03 (1.02–1.04)) is trivially above unity; and that even the hypothyroidism–dyslipidaemia association (1.29) is modest and an order of magnitude smaller than the effect of a decade of age (1.41). The Results now describe the correlations as 'statistically significant but negligible', the Methods state that interpretation emphasised effect sizes over p values, and the abstract characterises the correlations accordingly.

Changes made: Methods 'Statistical analysis' (page 8); Results 'Thyroid status and metabolic parameters' (page 10); Discussion, fourth paragraph (page 13); Abstract.

8 (Results)

Comment: Given the large sample size, did the authors consider evaluating the interaction between sex, age, and dyslipidaemia? I am unsure how clinically meaningful this would be, but from a statistical perspective it appears feasible to test in this dataset.

Response: We have added interaction and stratified analyses. The hypothyroidism × sex interaction (OR 1.06 (1.03–1.10)) and hypothyroidism × age interaction (1.07 (1.06–1.08) per 10 years) were both statistically significant, as the reviewer anticipated, but small: the adjusted OR was 1.29 (1.27–1.31) in women versus 1.20 (1.17–1.24) in men, and 1.24 (1.21–1.26), 1.35 (1.31–1.39) and 1.31 (1.26–1.36) at 18–39, 40–59 and ≥60 years. We share the reviewer's doubt about clinical meaning and say so explicitly in the Discussion.

Changes made: Methods (page 8); Results 'Sensitivity analyses' (page 11); Table 4 Panel B (page 21); Discussion (page 14).

9 (Discussion)

Comment: "Our very large, unselected clinical sample", "modest effect sizes are expected in an unselected population": while this phrasing may be accurate in that the study is based on electronic health records - it may give readers the impression that the results are representative of the general population. However, from what I can determine from the methods, these findings are based on individuals who approached primary care, which would typically be due to ill-health, pregnancy, or another concern. As such it may not be representative of the general population who did not attend

primary care (potentially less healthy), and it would be worth discussing in more detail the generalizability of the results.

Response: We agree entirely and have removed 'unselected' and 'population-representative' wherever they occurred (abstract bullets, Discussion). A new Generalisability section describes the tested population, the indication-driven nature of testing (symptoms, pregnancy screening, chronic-disease monitoring, follow-up of known thyroid disease), the likely direction of selection effects on prevalence and on the association, and the fact that the female predominance and its change over time reflect testing patterns. We state that the estimates describe the tested primary-care population of one province and that external validity depends on similar testing practices and iodine status.

Changes made: New 'Generalisability' section (page 14); 'Strengths and limitations of this study' bullets (page 3); Discussion second paragraph (page 12).

10 (Discussion)

Comment: "important covariates—including thyroid medication, iodine status, diet and comorbidity— were unavailable" I think it could be more directly stated that these are potential confounders, and therefore could impact the reported associations. As this is a significant limitation, the potential impact could be elaborated on - do the results risk underestimating/overestimating due to the lack of adjustment for these factors?

Response: We now state directly that these are potential confounders and discuss the direction of bias. Adiposity (unmeasured) is associated with both higher TSH and an atherogenic lipid profile and would bias the association away from the null; conversely, statin therapy in adults already known to have dyslipidaemia, and levothyroxine in those known to have hypothyroidism, would attenuate the observed association. The net direction is therefore uncertain. We quantify the sensitivity with an E-value of 1.52, meaning an unmeasured confounder associated with both exposure and outcome by a risk ratio of about 1.5 could fully account for the association; we name adiposity as a plausible candidate of that strength.

Changes made: Discussion, fourth paragraph (page 13); new 'Limitations' section (page 15); Table 4 (E-value row).

Reviewer 2 (Dr Jin-an Zhang, Jinshan Hospital of Fudan University)

General

Comment: ...several methodological and statistical issues — most notably severe residual confounding from adjusting only for age and sex while the dataset is heavily confounded by indication, an unexplained 9% sample attrition between the complete-assessment sample (1,318,078) and the regression sample (1,199,764), and selective reporting of the dyslipidaemia–thyroid dose–response — mean the manuscript as currently presented does not support its principal conclusion. Major revision is required.

Response: We thank the reviewer for a rigorous critique and have addressed each of the three concerns with new analyses rather than only new wording: (i) confounding is now quantified with an E-value and discussed with explicit direction-of-bias reasoning, and the principal conclusion has been reframed as an association independent of age and sex, modest in size, with causality explicitly not inferred; (ii) the 'attrition' is fully reconciled in a new flow diagram — it comprises adults without any lipid result on the index record and the hyperthyroid categories, which were never part of a hypothyroid-versus-euthyroid comparison — and a Missing data section has been added; (iii) all five categories are now reported for both outcomes with category-specific adjusted ORs, including

the inverse associations for the hyperthyroid categories, so that no part of the pattern is omitted. Details follow.

1

Comment: The multivariable model adjusts only for age and sex; the crude (1.32) → adjusted (1.30) shift is negligible, so residual confounding may fully explain the association. Please quantify via E-value/sensitivity analyses and state this limitation explicitly.

Response: We have calculated the E-value as requested. Because dyslipidaemia is common (prevalence 73.2%), the adjusted OR (1.29 (1.27–1.31)) was first converted to an approximate risk ratio by taking its square root (1.13), following VanderWeele and Ding; the resulting E-value is 1.52 for the point estimate and 1.50 for the lower confidence limit. We interpret this candidly: an unmeasured confounder associated with both hypothyroidism and dyslipidaemia by a risk ratio of about 1.5, beyond age and sex, could fully explain the association, and adiposity is a plausible candidate of that strength. We also note that the negligible crude-to-adjusted shift speaks only to confounding by age and sex, not to confounding in general. Additional sensitivity analyses (calendar-year adjustment, year-specific estimates, stratified and interaction models, category-specific models) are reported in Table 4 and Table 5. The limitation is stated explicitly in the abstract, the Strengths-and-limitations bullets, the Discussion and the new Limitations section, and the conclusion no longer claims independence from confounding.

Changes made: Methods 'Statistical analysis' (page 8); Results 'Sensitivity analyses' (page 11); Table 4 (E-value row, page 21); Discussion fourth paragraph (page 13); Limitations (page 15); new reference 21.

2

Comment: The complete thyroid assessment sample was n=1,318,078, but the regression sample included only n=1,199,764, a difference of 118,314 cases (≈9%) without any explanation. Please add a participant flow diagram and describe missing-data handling.

Response: A participant flow diagram has been added as the new figure 1, and a Missing data subsection has been added to Methods. The difference is now fully reconciled: of the 1,316,090 adults with a classified thyroid status, 88,172 had no lipid parameter on the index record (lipids are requested on clinical indication and are not part of every profile), leaving 1,227,918; of these, 27,634 had subclinical or overt hyperthyroidism and were not part of the hypothyroid-versus-euthyroid comparison, leaving 1,200,284 in the model. The same logic gives 1,263,138 for the dysglycaemia model. Every number is shown on the diagram, and complete-case handling is described and its assumption acknowledged in Limitations.

Changes made: New figure 1 and legend (page 23); Methods 'Missing data' (page 7); Results (pages 9–10); Table 4 n column.

3

Comment: In Figure 3, the abnormal blood glucose rate in the subclinical hyperthyroidism group (57.7%) was higher than that in the overt hypothyroidism group (55.4%), which did not conform to the simple dose-response pattern. It is more likely that this was caused by confounding factors such as the selection of detection indicators. Please provide additional discussion and necessary analysis.

Response: The reviewer's suspicion is correct, and the analysis we added confirms it. The hyperthyroid categories are markedly older than the other groups (mean age 52.0 and 55.0 years for subclinical and overt hyperthyroidism versus 43.8 years for euthyroid adults), and age is by far the strongest determinant of dysglycaemia (adjusted OR 2.01 per 10 years). In category-specific models

with euthyroid as reference, the univariable ORs for dysglycaemia of 1.47 (1.43–1.51) (subclinical hyperthyroidism) and 1.76 (1.68–1.86) (overt hyperthyroidism) fell to 0.92 (0.89–0.95) and 0.98 (0.92–1.04) after adjustment for age and sex — the crude excess is entirely an age effect, and subclinical hyperthyroidism is, if anything, inversely associated with dysglycaemia. We discuss this as consistent with confounding by indication (low TSH in older adults often reflects levothyroxine over-replacement or non-thyroidal illness rather than endogenous hyperthyroidism) and as an illustration of why unadjusted comparisons across categories should be interpreted cautiously. Figure 3 now carries significance markers, and its legend and the Results text no longer describe a simple dose–response across all five categories, only across the hypothyroid spectrum.

Changes made: Results 'Thyroid status and metabolic parameters' and 'Sensitivity analyses' (pages 10–11); Table 4 Panel C (page 21); Discussion fifth paragraph (page 13); figure 3 and legend.

4

Comment: STROBE checklist, flow diagram, sensitivity analyses, and a Generalisability paragraph are missing. Please add them.

Response: All four are now provided: a completed RECORD checklist incorporating every STROBE item, with page locations, is uploaded as a separate file (a STROBE checklist accompanied the original submission but we apologise if it was not visible to the reviewer); the flow diagram is the new figure 1; four sensitivity analyses are described in Methods and reported in Results, Table 4 and Table 5; and a Generalisability section has been added to the Discussion.

Changes made: RECORD_STROBE_checklist.docx; figure 1; Methods (page 8), Results (page 11), Tables 4–5; 'Generalisability' (page 14).

5

Comment: Abstract omits the univariable OR, dysglycaemia OR, and Spearman coefficients. Please add them.

Response: All three have been added to the abstract Results: the univariable OR for dyslipidaemia (1.31 (1.29–1.33)), the adjusted OR for dysglycaemia (1.06 (1.04–1.07)) and the range of Spearman coefficients (lipids 0.021 to 0.046; HbA1c -0.055), characterised as statistically significant but negligible in magnitude. The abstract also now mentions the E-value and the calendar-year result.

Changes made: Abstract 'Results' (page 2).

6

Comment: Figure 2 has no P value, and Figure 3 has no group significance markers.

Response: Both figures have been redrawn. Figure 2 shows the chi-square p value for the sex difference within each age group (brackets) and the overall p values for age and sex in the panel. Figure 3 shows significance markers for each category versus the euthyroid reference for both outcomes, with the key in the legend. All underlying p values are also given in Tables 2 and 4.

Changes made: Figures 2 and 3 and their legends (page 23).

7

Comment: Figure 1 is exactly the same as Table 1. It is recommended to redesign or delete Figure 1.

Response: Agreed. The previous figure 1 has been deleted and replaced by the participant flow diagram (which both reviewers and the Editor requested), so the number of figures remains four and every figure now adds information not available in the tables.

Changes made: New figure 1 and legend.

VERSION 2 - REVIEW

**Reviewer**	**2**
**Name**	**Zhang, Jin-an**
**Affiliation**	**Jinshan Hospital of Fudan University**
**Date**	**06-Sep-2026**
**COI**	

The manuscript has been substantively revised to address every prior concern with new analyses (E-value, calendar-year stratification, interaction/category-specific models, flow diagram) rather than only textual softening. The residual caveat on unmeasured confounding is honestly quantified. Suitable for publication.
